# Adult pilocytic astrocytomas: challenging the benign paradigm with surgical risks, recurrence dynamics, and molecular insights

**DOI:** 10.1007/s10143-025-03800-x

**Published:** 2025-09-19

**Authors:** Dorothea Mitschang, Helena Kleineidam, Felix Hinz, Felix Sahm, Andreas Unterberg, Sandro Krieg, Philip Dao Trong, Pavlina Lenga

**Affiliations:** 1 Department of Neurosurgery, Heidelberg University Hospital, Heidelberg, Germany; 2 Medical Faculty of Heidelberg University, Heidelberg, Germany; 3 Department of Neuropathology, Heidelberg University Hospital, Heidelberg, Germany; 4 Department of Neurosurgery, Vivantes Klinikum Neukölln, Berlin, Germany; 5 Department of Neurosurgery, University of Heidelberg, Im Neuenheimer Feld 400, Heidelberg, 69120 Germany

**Keywords:** Adult pilocytic astrocytoma, Infratentorial tumors, Recurrence, Ki-67, BRAF mutation, Surgical outcomes.

## Abstract

Purpose Adult pilocytic astrocytoma (APA) is rare and clinically distinct from pediatric counterparts. Despite generally favorable prognosis, recurrence rates vary significantly based on tumor characteristics and surgical approaches. Literature regarding the influence of tumor volume, location, and patient age on surgical outcomes and survival is limited and inconclusive. This study addresses these gaps, evaluating the combined impact of these variables on APA prognosis and management. Methods We retrospectively analyzed 32 adult patients with surgically treated APA at our institution (2014–2023), examining demographics, imaging, histology, and outcomes. Results Mean age was 35.8 years (SD 11.6); 59% were male. Median Karnofsky Performance Score (KPS) at admission was 90% (range 50–100%). Infratentorial tumors (56%) correlated significantly with lower KPS, increased cranial nerve deficits, cerebellar symptoms, hydrocephalus risk, prolonged operative time, higher CSF leaks (11%), and frequent revision surgeries. Gross total resection was achieved less frequently in infratentorial (33%) compared to supratentorial tumors (43%). Overall recurrence rate was 50%, strongly predicted by higher Ki-67 proliferation indices (*p* < 0.05), whereas resection extent alone lacked significant correlation. BRAF mutations occurred in only 38% of recurrent cases, highlighting APA’s distinct molecular profile. Five-year mortality was 6%, exclusively in High-Grade Astrocytoma with Piloid Features (HGAP). Conclusion Our findings challenge assumptions of benign clinical courses for APAs. Infratentorial tumors present increased surgical challenges and require tailored management. With recurrence rates of 50% and Ki-67 as a key prognostic marker, APA treatment demands personalized, biomarker-guided strategies beyond conventional surgical approaches.

## Introduction

Pilocytic astrocytoma (PA) is classically recognized as a pediatric tumor, representing approximately 25% of all childhood central nervous system (CNS) tumors [1]. Histologically, most pediatric PAs are World Health Organization (WHO) Grade 1, characterized by a generally indolent course and favorable long-term outcomes when adequately treated. Although PAs predominantly affect children, a subset of cases also arises in adults—an entity often referred to as APA — which accounts for approximately 1–2% of adult CNS tumors [1, 2].

Despite sharing a histological Grade 1 classification with pediatric tumors, APAs may exhibit distinct clinical and biological characteristics [1, 3, 4]. For instance, tumor location in adults is often infratentorial but can also be supratentorial, potentially affecting the feasibility of GTR and subsequent outcomes [2, 5, 6, 14]. GTR is widely regarded as the preferred surgical strategy, as it has been associated with reduced recurrence rates and improved survival; however, achieving this goal can be challenging due to the complex anatomy of infratentorial regions [1, 7, 8]. Additionally, the precise impact of tumor volume, tumor location, and patient age on long-term prognosis remains only partially understood, with existing literature offering limited and sometimes conflicting evidence.

Given these gaps, an improved understanding of the clinical behavior and optimal management strategies for APA is necessary. This single-center study therefore aims to (1) characterize the demographic and clinical profile of adult patients with PA, (2) evaluate the influence of surgical approaches—particularly the extent of resection—on survival and recurrence, and (3) assess the roles of tumor location, volume, and patient age in predicting outcomes. By synthesizing clinical data from a well-defined cohort and integrating existing evidence, we seek to clarify prognostic factors and guide treatment decisions for this understudied subgroup of low-grade gliomas.

## Methods

### Study design and data collection

We retrospectively evaluated the clinical and imaging data collected from our institution’s database between 2014 and 2023. This study was approved by the local ethics committee of our institution (reference S-005/2003, as of 31 st January 2003) and was conducted in accordance with the Declaration of Helsinki. The requirement for informed consent was waived owing to the retrospective nature of the study. Patients aged ≥18 years with histologically confirmed PA and available MRI were consecutively enrolled. The exclusion criteria were as follows: age < 18 years, other diagnosis than PA after histopathological workup. Patients were excluded if the required data including imaging were unavailable in their medical records.

### Patient characteristics

Electronic medical records were accessed to obtain patient demographics, comorbidities, American Society of Anesthesiologists (ASA) scores, Karnofsky performance score (KPS), body mass index (BMI), tumor location and volume, symptoms, histopathological findings, duration of surgery, peri- and postoperative complications, extent of tumor removal, length of hospital stay (LOS), intensive care unit (ICU) stay, postoperative imaging, readmission, reoperation, adjuvant therapy, recurrence and mortality. Tumor volume was assessed by manual volumetry of either contrast enhancing mass or in non-contrast enhancing tumors of FLAIR-hyperintense mass in Brain Lab cranial planning software suite. Extent of tumor removal was assessed via postoperative MRI and divided by gross total resection or subtotal resection, as evaluated by two experienced neurosurgeons (PDT, PL). Follow-up data as scheduled for clinical and radiologic routine examinations was accessed via our institutions medical database.

### Statistical analysis

Categorical variables are presented as numbers and percentages. Continuous variables are presented as mean and standard deviation. Baseline characteristics, duration of surgery, perioperative and postoperative complications, LOS, ICU stay, readmissions, reoperations, and mortality were compared group wise using independent t-tests for continuous variables and fishers exact test for categorical variables. In the second-stage analysis correlation between e.g. age or extent of resection and recurrence was calculated by Spearman’s correlation. Statistical significance was set at a p-value ≤ 0.05.

## Results

### Demographic and clinical characteristics of adult patients with pilocytic Astrocytoma

In total, 32 adult patients (≥ 18 years) diagnosed with PA between 2014 and 2023 were included in this study. The mean age at diagnosis was 28.2 years (SD 15.8), with the majority (81%, *n* = 26/32) being younger than 40 years. A slight male predominance was observed (59%, *n* = 19/32). Most patients presented in good clinical condition, with a median KPS of 90% (range, 50–100%) at admission, and 88% (*n* = 28/32) reported no more than two symptoms. The most frequent presenting complaints were new-onset headaches (50%, *n* = 16 = 32), symptoms of elevated intracranial pressure (47%, *n* = 15/32), including nausea and vomiting, and visual disturbances (25%, *n* = 8/32). Given the predominance of infratentorial lesions (56%, *n* = 18/32), posterior fossa symptoms such as ataxia (19%, *n* = 6/32) and vertigo (9%, *n* = 3/32) were more prevalent than motor (13%, *n* = 4/32) or sensory deficits (3%, *n* = 1/32). The median preoperative tumor volume was 10.1 cm³ (range, 0.8–95.0 cm³, IQR 3.5). Baseline characteristics are depicted in Table 1.

**Table 1:** Demographic and clinical characteristics of adult patients with pilocytic astrocytoma

	*n* = 32	%
Sex		
Male	19	59
Female	13	41
Age at diagnosis (years)		
Mean (SD)	28.2 (15.8)	
≤ 40	26	81
40–49	2	6
50–69	3	9
≥ 70	1	2
KPS at presentation		
Median (range)	90 (50–100%)	
BMI at presentation		
Mean (SD)	25.1 (11.6)	
Symptoms at presentation		
New-onset headache	16	50
Visual disturbances	8	25
Symptoms of elevated ICP	15	47
Vertigo	3	9
Motoric	4	13
Sensory	1	3
Seizures	2	6
Ataxia	6	19
Speech disturbance	1	3
Symptomatic burden at presentation		
≤ 2 symptoms	28	88
> 2 symptoms	4	13
Tumor location		
supratentorial	14	44
infratentorial	18	56
Median Tumor volume in cc (range, IQR)	10.1 (0.9–95.3, 3.5)	

Abbreviations: *KPS* Karnofsky performance score, *BMI b*ody mass index, *ICP i*ntracranial pressure,* IQR* interquartile range

### Surgical characteristics and clinical course

GTR was achieved in 44% (*n* = 14/32) of cases, while 34% (*n* = 11/32) underwent subtotal resection (STR) due to tumor infiltration into eloquent brain regions (Table 2). In 22% (*n* = 7/32) of cases, only biopsy procedures were feasible due to anatomical constraints, such as brainstem or thalamic involvement. One patient (3%) who was initially intended for GTR required revision surgery after postoperative imaging revealed residual tumor. Neither tumor location (supratentorial vs. infratentorial) nor preoperative tumor volume significantly correlated with resectability. Postoperatively, patients remained in the ICU for a mean of 2.4 days (SD 7.4) and had a mean total hospitalization of 11.9 days (SD 11.4). No in-hospital or 90-day mortality was observed, and the overall mortality rate by 2023 was 6% (*n* = 2/32), both of whom had HGAP tumors. Adjuvant therapy was required in 43% (*n* = 14/32) of cases, with 28% (*n* = 9/32) receiving chemotherapy and 16% (*n* = 5/32) undergoing radiotherapy. Most (97%, *n* = 31/32) patients maintained or returned to a functional status similar to their preoperative baseline, with a median KPS of 90% (range 50%−100%) at discharge.

**Table 2:** Surgical characteristics and clinical course of adult patients admitted with pilocytic astrocytoma

	*n* = 32	%
Extent of Tumor removal		
GTR	14	44
STR	11	34
Biopsy	7	22
Postoperative imaging		
GTR	14	44
STR	11	34
Length of ICU Stay (days), mean (SD)	2.4 (7.4)	
Length of hospital stay (days), mean (SD)	11.9 (11.4)	
30-day re-admission	4	13
In-hospital mortality	0	0
90-day mortality	0	0
Total mortality	2	6
Adjuvant therapy	14	44
Chemotherapy	9	28
Radiation	5	16
KPS at discharge		
Median (range)	90 (50–100%)	

Abbreviations: *GTR* gross total resections, *STR* subtotal resections, *VP-Shunt v*entriculoperitoneal shunt, *ICU* intensive care unit, *KPS* karnofsky performance score, *SD* standard deviation

### Postoperative complications

Surgical complications occurred in 28% (*n* = 9/32) of patients, with new postoperative neurological deficits observed in 16% (*n* = 5/32) (compare to Table 3.). Most were transient, except for a single case of persistent vertical oculoparesis. Sensory disturbances were the most common (6%, *n* = 2/32) deficits. CSF fistulas developed in 6% (*n* = 2/32), necessitating revision surgery for dural repair. Additionally, 16% (*n* = 5/32) of patients with infratentorial tumors developed postoperative hydrocephalus. Non-surgical complications occurred in 22% (*n* = 7/32), with seizures and urinary tract infections (6%, *n* = 2/32 each) being the most frequent, followed by isolated cases of cardiac events, respiratory infections, and hypernatremia.

**Table 3:** Postoperative surgery-related and non-surgery-related complications of adult patients with pilocytic astrocytoma which received an operative treatment

	*n* = 32	%
Surgery-related complications	9	28
Wound infection	1	3
CSF-fistula	2	6
Meningitis	1	3
New postoperative deficit	5	15
* Status epilepticus*	1	3
* Sensory deficits*	2	6
* Vertical oculoparesis*	1	3
* Speech disturbance*	1	3
Revision surgery	3	9
Dural plastic	2	6
Resection of tumor remnant	1	3
Postop. Hydrocephalus with VP-Shunt	5	16
Non-surgery-related complications	7	22
Cardiac	1	3
Urinary infection	2	6
Respiratory	1	3
Seizures	2	6
Hypernatremia	1	3

Abbreviations: *CSF* Cerebrospinal Fluid, *VP* ventriculoperitoneal

### Adjuvant therapy

Adjuvant therapy was required in 43% (*n* = 14/32) of cases. Chemotherapy was administered to 28% (*n* = 9/32) of patients and included temozolomide (*n* = 1), CCNU (lomustine, *n* = 1), targeted therapies such as dabrafenib plus trametinib (*n* = 1), tovorafenib (*n* = 1), and unspecified agents (*n* = 1). Additionally, one patient underwent implantation of a Rickham reservoir for cyst management, and another received palliative care. Five patients (16%, *n* = 5/32) received radiotherapy, although specific details regarding radiation modalities or concurrent use with chemotherapy were unavailable. Furthermore, 6 patients were managed with a watch-and-wait strategy without immediate active treatment.

### Histopathological findings and recurrence rates

Histopathological analysis confirmed that 91% (*n* = 29/32) of cases were WHO Grade 1 tumors, while 9% (*n* = 3/32) were HGAP (refer to Table 4.). The mean Ki-67 proliferation index was 4.7% (SD 3.7), consistent with the typically indolent behavior of PA. However, higher Ki-67 values at initial diagnosis correlated significantly with recurrence risk (Spearman *r* = 0.3717, *p* = 0.04).

**Table 4:** Histological findings of adult patient with pilocytic astrocytoma

	*n* = 32	%
Histological Grading		
WHO Grad 1	29	91
HGPA^*^	3	9
Ki-67 proliferation index at first diagnosis		
mean (SD)	4.7 (3.7)	

Abbreviations: *WHO* world health organization, *BRAF* proto-oncogene B-Raf., *SD* standard deviation, *HGPA* high-grade astrocytoma with piloid features, * following molecular analysis

Tumor recurrence occurred in 50% of cases (*n* = 16/32), with a mean time to recurrence of 19.4 months (SD 15.2). The mean age at recurrence was 36.7 years (SD 20.2) (refer to Table 5.). Among recurrent cases, 50% (*n* = 8/16) required reoperation, with WHO Grade 1 histology confirmed in seven cases and HGAP in one. The remaining cases were managed conservatively, with 38% (*n* = 6/16) undergoing watchful waiting and 25% (*n* = 4/16) receiving chemotherapy. One patient with HGAP in the thalamus received palliative care. Notably, neither GTR at initial surgery nor initial tumor volume correlated with recurrence risk. However, higher Ki-67 at diagnosis correlated with a greater likelihood of recurrence, despite a paradoxically lower Ki-67 on repeat histology (mean 3.0%, SD 1.4) compared to initial specimens (mean 4.7%, SD 3.7). Among patients with documented recurrence and available Ki-67 data (*n* = 8), the mean Ki-67 index was 4.7% (SD 3.7), suggesting an association between elevated proliferative activity and recurrence. However, Ki-67 values were not available for non-recurrent cases, precluding formal statistical comparison between groups. As such, no Mann-Whitney U test or regression analysis could be conducted to quantify the predictive value of Ki-67 in this cohort. Additionally, BRAF mutations were identified in 38% (*n* = 3/16) of recurrent tumors. Due to inconsistent testing for BRAF mutations in the initial pathological specimens we did not perform an analysis and comparison of BRAF mutations in initial and recurrent tumors.

**Table 5:** Recurrence rates and histological findings at recurrence

	*n* = 32	%
Recurrence	16	50
Mean Time to recurrence in months (SD)	19.4 (15.2)	
Mean Age at recurrence (SD)	36.7 (20.2)	
Treatments at first recurrence		of total recurrences *n* = 16
Primary resection	8	50
Radiotherapy	0	0
Chemotherapy	4	25
Watch and Wait	6	38
Palliative	1	6
Histology at recurrence		
Grading		
WHO 1	7	
WHO 2	0	
WHO3	1	
Mean Ki-67% (SD)	3.0 (1.4)	
BRAF-Status at recurrence		
Positive	3	38
Negative	5	63
Histological progression	0	0

Abbreviations: *WHO* world health organization, *BRAF* proto-oncogene B-Raf, *SD* standard deviation

### Comparison of supra- and infratentorial tumor location

Infratentorial tumors were more frequently associated with cranial nerve deficits (CND) (36% vs. 17%, *p* = 0.005), posterior fossa symptoms (56% vs. 14.3%, *p* = 0.008), and preoperative hydrocephalus (56% vs. 21%, *p* = 0.001) (compare to Table 6.). Preoperative KPS was lower in the infratentorial group (85% vs. 90%, *p* = 0.047), and median tumor volume was larger (11.6 cm³ IQR 3.8 vs. 9.9 IQR 4.1 cm³, *p* = 0.049). Revision surgeries were more common in infratentorial tumors (39% vs. 21%, *p* = 0.043), primarily due to postoperative CSF fistulas. Although age at recurrence did not differ, patients who underwent STR tended to experience earlier recurrence.

**Table 6:** Comparison of supra- and infratentorial tumor locations

	Supratentorial (*n* = 14)	Infratentorial (*n* = 18)	*p*-value
Age (years), mean (SD)	28.1 (2.1)	29.3 (1.2)	0.783
Sex, n (% of group)			0.928
Male	5 (36%)	10 (56%)	–
Female	9 (64%)	8 (44%)	–
Symptoms, n (% of group)			
CND	3 (17%)	5 (36%)	**0.005**
MD	12 (86%)	15 (83%)	0.835
Cerebellar	2 (14%)	10 (56%)	**0.008**
Hydrocephalus	3 (21%)	10 (56%)	**0.001**
KPS preoperative, mean (SD)	91 (10.7)	85 (11.5)	**0.047**
Tumor volume (cm²), median (range, IQR)	9.9 (1.3–68.3, 3.8)	11.6 (0.9–95.1, 4.1)	**0.049**
Extent of resection, n (% of group)			0.889
GTR	6 (43%)	6 (33%)	–
STR	8 (57%)	11 (61%)	–
Duration of surgery (min), mean (SD)	270.0 (51)	273.6 (35)	0.598
Complications	–	–	0.446
Revision surgery, n (% of group)	3 (21%)	7 (39%)	**0.043**
Age at recurrence (years), mean (SD)	31.0 (41.0)	37.0 (18.3)	0.099

Abbreviations: *SD* standard deviation, *CND* cranial nerve deficit, *MD* motor deficit, *KPS* karnofsky performance status, *GTR* gross total resection, *STR* subtotal resection, *IQR* interquartile range

## Discussion

PAs are the most common pediatric brain tumors, yet their occurrence in adults remains rare and poorly understood. Unlike their pediatric counterparts, adult PAs often present with distinct clinical challenges, potentially more aggressive behavior, and higher recurrence rates [1, 6]. Although classified as WHO Grade 1 tumors, adult PAs exhibit a heterogeneous clinical course, and the optimal management strategies remain a subject of debate due to the scarcity of large, dedicated adult PA studies [1, 2, 9, 10]. Our study contributes a detailed, location-specific analysis of adult PA, revealing that infratentorial tumors are associated with a higher symptom burden, increased surgical complexity, more frequent complications, and greater need for revision surgery. Additionally, we provide novel insights into recurrence risk, molecular features, and surgical outcomes, emphasizing the clinical and prognostic implications of these factors.

### Clinical presentation: anatomical constraints and functional outcomes

Widhalm et al. reported that adult pilocytic astrocytomas (APA) are roughly evenly split between supratentorial and infratentorial sites (≈ 59% vs. 41%) [11], with a substantial subset of supratentorial tumors involving midline structures (e.g. ~34% in the optic pathway/hypothalamus) [12]. They quantified distinct clinical presentations: headaches were the most common symptom (65% of cases), followed by visual disturbances (~ 35%), whereas seizures were relatively infrequent (~ 9%) [13]. This aligns with the notion that supratentorial APAs tend to produce focal deficits or seizures, while infratentorial lesions more often manifest with signs of raised intracranial pressure (headache, nausea/vomiting) and cerebellar dysfunction. In Widhalm’s series, tumor location per se did not dramatically alter long-term prognosis – instead, extent of resection was paramount, as virtually all tumor recurrences (~ 15% overall) occurred in cases of subtotal resection, regardless of supra- or infratentorial location [14]. Subsequent studies have reinforced that anatomical localization influences surgical risk and outcomes indirectly: adult PAs frequently arise in surgically challenging areas (e.g. insular or suprasellar region), which often limits safe resectability [12]. Indeed, deep-seated tumors (thalamo–brainstem region) achieve far lower gross-total resection rates (~ 14% vs. 75% in more accessible locations) and are associated with markedly higher progression risk [15]. Our observations are in agreement with earlier investigations, notably the extensive study by Widhalm et al. [11] which verified that the anatomical location played an important role in determining clinical manifestations and surgical difficulty between APAs. Although previous studies have demonstrated that supratentorial and infratentorial APAs differ, our study adds quantitative detail to that link by associating tumor location with perioperative functional deficits and larger tumor volumes at diagnosis, raising the possibility of delayed clinical presentation. These results demonstrate the importance of tumor site in consideration of risk profile and therapeutic management in the management of APA.

### Surgical complexity: increased risks and the role of location

Surgical resection remains the gold standard for PA treatment, yet our study highlights the substantial challenges posed by infratentorial tumor location. The impact of tumor location on operative duration, complication rates, and revision surgery frequency is a significant aspect of our study. Infratentorial resections in our cohort were associated with significantly longer operative times, consistent with studies highlighting the technical demands of posterior fossa surgery [7, 8, 16]. Given the delicate neurovascular structures at risk, surgeons often employ a slower and more meticulous dissection strategy to avoid iatrogenic deficits, which may prolong surgery but is necessary to preserve neurological function. Additionally, our data underscore the higher frequency of revision surgeries in infratentorial tumors, particularly in cases complicated by postoperative CSF leaks and hydrocephalus. The high incidence of postoperative CSF leakage in infratentorial tumors (11%) reflects the inherent vulnerability of the posterior fossa dura, which is thinner and less compliant than its supratentorial counterpart [8]. The dynamic nature of CSF flow in this region, combined with negative intracranial pressure fluctuations, creates an environment where even minor dural defects can result in persistent CSF leakage, necessitating surgical repair. These findings align with the 3–15% rate of CSF leaks in posterior fossa surgery reported in systematic reviews [7], reinforcing the importance of meticulous intraoperative closure techniques, dural reinforcement, and CSF diversion strategies in high-risk cases. The use of autologous pericranial grafts, synthetic dural substitutes, and fibrin-based sealants has been widely recommended to enhance closure integrity [17], and our data suggest that such measures should be routinely considered in infratentorial cases. Hydrocephalus remains a well-recognized postoperative complication of posterior fossa tumors, yet our findings indicate that its incidence in APA has been underappreciated. We observed that all cases of postoperative hydrocephalus occurred in infratentorial tumors, likely due to the obstructive nature of fourth-ventricle involvement. Given these findings, prophylactic CSF diversion, including perioperative lumbar drainage or early ventriculostomy, should be considered in cases where preoperative imaging suggests high-risk tumor positioning [6–8, 17].

### Recurrence patterns and the emerging role of Ki-67 as a prognostic marker

Our findings suggest a potential link between higher Ki-67 indices and recurrence in APA, aligning with previous studies that highlight proliferation markers as prognostic indicators. However, due to the retrospective nature of the data, Ki-67 values were only available in a subset of recurrent patients. The absence of Ki-67 measurements in non-recurrent cases limited our ability to perform statistical tests or model recurrence risk more rigorously. This reinforces the need for prospective studies with consistent biomarker acquisition.

The low prevalence of BRAF mutations in recurring tumors in our cohort (38%) further supports the hypothesis that adult and pediatric PAs differ at a molecular level, reinforcing findings by Theeler et al. [18]. While BRAF-KIAA1549 fusions and BRAF^V600E mutations are well-characterized in pediatric PAs, their prognostic significance in adult tumors remains unclear. Given the success of BRAF/MEK inhibitors in pediatric PA clinical trials, future studies should explore whether a subset of APAs may respond to targeted therapy. The absence of routine molecular profiling in current APA management represents a significant knowledge gap, underscoring the need for next-generation sequencing studies to refine our understanding of APA biology.

Our finding of 50% of APA recurrence in adults is much higher than that previously reported in the literature, which included the report by Muhsen et al. [12] and Bond et al. [1] with a recurrence rate of 7 and 13%, respectively. This discrepancy requires careful reflection. Muhsen et al. only enrolled 15 adults APA patients, and the median follow up was as short as 11 months, and recurrence might be underestimated in this cohort because of limited time of follow up to survey late recurrences, which are common disease manifestation of APA, since the disease is usually indolent. Furthermore, in their series 50% of patients underwent GTR or near-total resection of mainly surgically accessible tumors. Likewise, Bond et al., who has followed 46 adults for a longer period (approx. 6.5 years), have described recurrence in 13% of patients with GTR performed in a little more than half of their patients. The systematic review incorporated in their study demonstrated even higher pooled recurrence rate 31%, emphasizing the variation in the recurrent outcomes owing to surgical and anatomical factors. In comparison, our series reflects a population of patients at a dedicated tertiary referral center, with tumors often found in technically difficult infratentorial and deep brain sites, which prohibits an approach of GTR (44% in our series). In addition, our strict definition of the recurrence, involving an established imaging progression and clinical indication for re-intervention, and the long follow-up (up to 9 years) probably led to a more reliable and maybe higher detection of recurrences. Therefore, instead of contradicting data, our higher rate of recurrence probably is the result of a higher methodological stringency, differences in tumor location, collecting of more complex cases, reflecting the extensive diversity of APA and drawing attention to the necessity of applying personalized therapeutic approaches in these particular group of patients.

Recent advancements in molecular diagnostics have reshaped our understanding of PAs and their high-grade counterparts, revealing that many tumors previously classified as “anaplastic pilocytic astrocytoma” are, in fact, High-Grade Astrocytoma with Piloid Features (HGAP) [19–21]. Unlike conventional PAs, HGAP exhibits a more aggressive clinical course, with recurrence and progression rates that place its prognosis between classic PA and IDH-wildtype glioblastoma [19–21]. Our study highlights this aggressive potential, as both fatalities in our cohort were due to progressive disease, likely reflecting an underlying HGAP biology rather than a conventional “malignant transformation” of PA. Despite multimodal therapy, HGAP frequently recurs within 1–2 years, and its five-year survival (~ 50%) is significantly worse than that of classic PA [19–21]. Notably, traditional histologic markers (e.g., necrosis, mitotic index) are unreliable predictors of outcome in HGAP, making DNA methylation profiling essential for accurate classification [19–21]. This approach identifies key molecular drivers—frequent MAPK pathway alterations (e.g., NF1 loss, BRAF fusions, FGFR1 mutations) and CDKN2A/B deletions—which contribute to rapid progression and treatment resistance [19–21]. Clinically, recognizing HGAP is critical, as it offers opportunities for targeted therapy beyond standard radiochemotherapy. MEK inhibitors may be beneficial in NF1- or BRAF-driven cases, FGFR inhibitors for FGFR1-fusion tumors, and mTOR inhibitors in NF1-associated disease [19–21]. Though investigational, these approaches underscore the need for molecular surveillance in any PA with atypical progression. Our findings emphasize that histologic assessment alone may misclassify these tumors, and precise molecular characterization is essential for risk stratification and therapy selection. By integrating these insights into clinical practice, we can better tailor treatment strategies and improve patient outcomes.

### Rationale for limited use of radiotherapy in our adult APA cohort

Although Bond et al. [1] include radiotherapy (RT) among primary and salvage options for adult pilocytic astrocytoma (APA), evidence for its benefit in this population remains inconsistent and, at times, unfavorable. In their institutional series, only three of forty-six patients received up-front RT, and two of those three experienced progression, contributing to their overall 13% recurrence rate; moreover, in the pooled analysis of 254 cases, progression was significantly driven by subtotal resection rather than by omission of adjuvant RT [22]. Subsequent contemporary studies have raised additional concerns. A population-based analysis of adults undergoing subtotal resection found that postoperative RT was associated with a higher hazard of death (HR 1.6) compared with observation, suggesting that RT may not translate into a survival advantage after incomplete resection [23]. Likewise, a molecularly annotated Mayo Clinic cohort reported that adjuvant RT correlated with shortened progression-free survival (median 22 months vs. > 60 months in non-irradiated cases; *P* = 0.004) and did not improve overall survival [18]. While an earlier single-institution study demonstrated longer progression-free survival with immediate RT (5-year PFS 91% vs. 42% under observation), overall survival remained identical because salvage therapies—including re-irradiation and repeat resection—were effective at recurrence, thereby negating an early-RT advantage [24]. Balancing these mixed oncologic outcomes against well-documented late toxicities—neurocognitive decline, endocrine dysfunction, and, in rare instances, radiation-induced secondary malignancies—our multidisciplinary board adopted a highly selective RT policy. Infratentorial and deep midline APAs in our series were managed with maximal safe resection followed by close MRI surveillance; RT was reserved for unequivocal radiographic or symptomatic progression not amenable to repeat surgery or emerging targeted therapies (e.g., BRAF/MEK or FGFR inhibition). This strategy explains the 16% utilization rate in our cohort yet remains consistent with contemporary data indicating no clear survival benefit—and potential harm—of indiscriminate adjuvant irradiation in adults with APA. Prospective, molecularly stratified trials are needed to delineate which subgroups, if any, derive net benefit from early RT in the era of precision oncology.

### Comparative insights into adult vs. Pediatric pilocytic Astrocytoma

The more aggressive clinical course of pilocytic astrocytoma (PA) in adults, as observed in our cohort, may be attributed to several key molecular, anatomical, and diagnostic factors. Molecular profiling studies reveal that adult PAs are less frequently driven by the canonical *KIAA1549–BRAF* fusion common in pediatric cases (present in up to 98% of pediatric PAs [12, 25], and instead show a more diverse range of MAPK pathway alterations, including *BRAFV600E* mutations and *FGFR1* aberrations [26, 27], which are linked to poorer progression-free survival, particularly when co-occurring with *CDKN2A/B* deletions [28, 29]. These genetic differences suggest a higher intrinsic malignant potential in adult PAs. Anatomically, pediatric tumors often arise in the cerebellum, allowing for curative resection, whereas adult tumors are more frequently located in deep-seated or eloquent brain regions, such as the thalamus or brainstem, where gross-total resection is frequently not feasible [30, 31]. This contributes to higher rates of residual tumor and recurrence in adults [32]. Additionally, adult PAs display greater variability in proliferative indices, with Ki-67 values often exceeding those seen in pediatric tumors and sometimes approaching anaplastic thresholds [33]. These biological patterns are paralleled by diagnostic delays in adults due to atypical symptoms or misattribution to non-neoplastic causes [34]. Together, these factors reflect that adult PAs—while histologically similar—are clinically and biologically distinct from their pediatric counterparts, warranting a tailored diagnostic and therapeutic approach that includes early molecular testing, aggressive surgical planning when feasible, and consideration of targeted therapies in high-risk or recurrent cases.

## Limitations

Despite being one of the most comprehensive studies on APAs to date, several limitations must be acknowledged. First, the retrospective nature of our study inherently introduces selection bias and limits the granularity of certain clinical variables. While we meticulously analyzed preoperative and postoperative outcomes, a prospective, multicenter study with standardized surgical protocols and follow-up criteria would provide a more definitive understanding of APA behavior. Second, although our cohort is one of the largest single-center series of APA patients, the overall sample size remains relatively small given the rarity of the disease. Larger datasets are necessary to validate our findings on surgical risks, recurrence patterns, and molecular prognosticators, especially in defining the role of Ki-67 and BRAF status in recurrence risk stratification. A major limitation of our analysis was the incomplete documentation of Ki-67 indices, particularly in non-recurrent cases. While elevated Ki-67 was observed in recurrent tumors, the lack of corresponding data in recurrence-free patients precluded statistical comparisons or predictive modeling. Future studies should incorporate routine immunohistochemical profiling to facilitate robust prognostic evaluation. Third, our study, while incorporating histopathological data, lacks full genomic profiling especially regarding tumor specimens after initial operation, limiting our ability to draw firm conclusions about the molecular underpinnings of APA. Future studies should integrate whole-exome sequencing and transcriptomic analysis of primary and recurrent tumors to identify potential therapeutic targets and biomarkers for recurrence risk. The low frequency of BRAF mutations in adult PAs compared to their pediatric counterparts raises intriguing biological questions about alternative oncogenic drivers, which remain largely unexplored. Fourth, the absence of long-term neurocognitive and quality-of-life assessments is a notable gap. Given the significant postoperative morbidity in infratentorial cases, including cranial nerve deficits and hydrocephalus, future research should focus on functional outcomes, long-term rehabilitation, and quality-of-life measures, particularly in patients undergoing revision surgeries. Fourth, due to the retrospective nature of this study, specific details regarding advanced neurosurgical techniques, such as awake craniotomy or intraoperative white matter tracking, were not uniformly recorded or analyzed. Future prospective studies should explicitly document these operative techniques to evaluate their impact on clinical outcomes and recurrence in adult pilocytic astrocytoma. In addition, the retrospective study design resulted in incomplete details regarding specific adjuvant treatment modalities, radiation types, chemotherapy regimens, and the rationale behind selecting watch-and-wait strategies. This lack of detail limits our ability to assess precisely the influence of different adjuvant therapeutic approaches on recurrence and clinical outcomes. Finally, while we demonstrate that extent of resection alone may not be the sole determinant of recurrence, the impact of adjuvant therapy in APA remains uncertain. There is currently no consensus on the role of radiotherapy, chemotherapy, or targeted therapies in adult patients. Clinical trials evaluating adjuvant therapy in high-risk APA subgroups—such as those with STR, elevated Ki-67, or molecularly aggressive profiles—are urgently needed.

## Conclusions

Our study underscores that adult pilocytic astrocytomas, particularly infratentorial tumors, present distinct surgical and clinical challenges including increased risks of hydrocephalus and neurological deficits. Elevated Ki-67 indices may predict recurrence more reliably than extent of resection alone, highlighting the importance of molecular markers for prognosis. Given the relatively low incidence of BRAF mutations in adults compared to pediatric cohorts, APA likely represents a biologically distinct entity, reinforcing the need for personalized, biomarker-driven therapeutic approaches. Future investigations integrating detailed molecular profiling and targeted therapies are essential to optimize management and improve outcomes in adult APA patients.

## Data Availability

No datasets were generated or analysed during the current study.

## Declarations

### Clinical trial number

Not applicable. This is a retrospective monocentric study approved by the local ethics committee (reference S-005/2003), as mentioned above.
